# Green, Blue, Brown, Gray? Nature exposure and cognitive function among aging Mexican Americans

**DOI:** 10.1002/alz.091919

**Published:** 2025-01-09

**Authors:** Hala J Darwish, Emily Hokett, Kayla N Tureson, Alexa C Allan, Jessica M Finlay, Noah J Webster

**Affiliations:** ^1^ University of Michigan, Ann Arbor, MI USA; ^2^ Columbia University, New York, NY USA; ^3^ University of Southern California, Los Angeles, CA USA; ^4^ The Pennsylvania State University, University Park, PA USA

## Abstract

**Background:**

Studies link nature exposure to better cognitive health outcomes. However, little is known about which types or ‘how much’ of nature is needed for health benefits. Studies often lack diverse aging populations and have small sample sizes.

**Method:**

Our study investigated the relationship between land cover types (e.g., water, green, developed, or barren) and cognitive function (Mini Mental Status Exam) in 2,069 participants from the Wave 5 Hispanic Established Population for the Epidemiological Study of the Elderly. Linear mixed‐effect models adjusted for individual (e.g., age) and area‐level (e.g., poverty) covariates were used.

**Result:**

The mean age was 81.9±5.2, 61.5% women, and the mean years of education was 4.9±4.0. The median MMSE score was 22, range of 0‐30. Latent Profile Analysis of land cover types revealed four distinct classes. Individuals living in less developed, green‐mix areas had higher cognitive scores, particularly among women.

**Conclusion:**

Environments with a mix of nature *and* access to services, amenities, and other people positively impact cognition in aging Mexican American women.

